# Retraction Note: The diagnostic nomogram of platelet-based score models for hepatic alveolar echinococcosis and atypical liver cancer

**DOI:** 10.1038/s41598-020-73758-x

**Published:** 2020-09-29

**Authors:** Qiancheng Du, Yanyan Wang, Shihao Guan, Chenliang Hu, Mengxuan Li, Ling Zhou, Mengzhao Zhang, Yichong Chen, Xuepeng Mei, Jian Sun, Ying Zhou

**Affiliations:** 1 Department of General Surgery, Shanghai Fourth People’s Hospital Affiliated To Tongji University School of Medicine, Shanghai, 200081 China; 2 Department of Hematology, Fuyang Hospital of Anhui Medical University, Fuyang, 236000 China; 3 Department of Plastic Surgery, The Qingpu Branch of Zhongshan Hospital Affiliated To Fudan University, Shanghai, 201700 China; 4 Department of Hepatopancreatobiliary Surgery, The Affiliated Hospital of Qinghai University and Qinghai Province Key Laboratory of Hydatid Disease Research, Qinghai University, Xining, 81000 China; 5 The Graduate School, Qinghai University, Xining, 810000 China

Retraction of: *Scientific Reports* https://doi.org/10.1038/s41598-019-55563-3, published online 18 December 2019

The Authors have retracted this Article.

In the original Article, the Authors reported that low platelet level in HAE patients and high platelet level in hepatocellular carcinoma patients are caused by the differences between these conditions. This did not account for the potential confounding factor that HAE combined with hepatitis B has a low degree of liver cirrhosis whereas hepatocellular carcinoma combined with hepatitis B has a high degree of liver cirrhosis, and so the platelet difference may be reflective of the different degrees of cirrhosis. Since the study did not contain non-hepatitis patients as controls, it was not designed to distinguish between these factors. Additionally, the Authors chose incorrect parameters as input into Lasso regression model, which affects the outcome of the analysis presented in Figure 3. Overall, the Authors cannot guarantee the validity of the results and the conclusions reported in this Article.

All Authors agree with the retraction and its wording.

